# Blending and Characteristics of Electrochemical Double-Layer Capacitor Device Assembled from Plasticized Proton Ion Conducting Chitosan:Dextran:NH_4_PF_6_ Polymer Electrolytes

**DOI:** 10.3390/polym12092103

**Published:** 2020-09-16

**Authors:** Shujahadeen B. Aziz, Mohamad A. Brza, Iver Brevik, Muhamad H. Hafiz, Ahmad S.F.M. Asnawi, Yuhanees M. Yusof, Rebar T. Abdulwahid, Mohd F.Z. Kadir

**Affiliations:** 1 Hameed Majid Advanced Polymeric Materials Research Lab., Physics, College of Science, University of Sulaimani, Qlyasan Street, Kurdistan Regional Government, Sulaimani 46001, Iraq; rebar.abdulwahid@univsul.edu.iq; 2 Department of Civil engineering, College of Engineering, Komar University of Science and Technology, Kurdistan Regional Government, Sulaimani 46001, Iraq; 3 Manufacturing and Materials Engineering Department, Faculty of Engineering, International Islamic University of Malaysia, Kuala Lumpur 50603, Malaysia; mohamad.brza@gmail.com; 4 Department of Energy and Process Engineering, Norwegian University of Science and Technology, N-7491 Trondheim, Norway; 5 Institute for Advanced Studies, University of Malaya, Kuala Lumpur 50603, Malaysia; hafizhamsan93@gmail.com; 6 Chemical Engineering Section, Universiti Kuala Lumpur, Malaysian Institute of Chemical & Bioengineering Technology (UniKL MICET), Alor Gajah 78000, Malacca, Malaysia; asyafiq.asnawi@s.unikl.edu.my (A.S.F.M.A.); yuhanees@unikl.edu.my (Y.M.Y.); 7 Department of Physics, College of Education, University of Sulaimani, Old Campus, Kurdistan Regional Government, Sulaimani 46001, Iraq; 8 Centre for Foundation Studies in Science, University of Malaya, Kuala Lumpur 50603, Malaysia; mfzkadir@um.edu.my

**Keywords:** chitosan-dextran blend electrolyte, ammonium hexafluorophosphate, glycerol, XRD analysis, electrochemical impedance spectroscopy study, electrochemical double-layer capacitor device

## Abstract

This research paper investigates the electrochemical performance of chitosan (CS): dextran (DX) polymer-blend electrolytes (PBEs), which have been developed successfully with the incorporation of ammonium hexafluorophosphate (NH_4_PF_6_). X-ray diffraction (XRD) analysis indicates that the plasticized electrolyte system with the highest value of direct current (DC) ionic conductivity is the most amorphous system. The glycerol addition increased the amorphous phase and improved the ionic dissociation, which contributed to the enhancement of the fabricated device’s performance. Transference number analysis (TNM) has shown that the charge transport process is mainly by ions rather than electrons, as *t*_ion_ = 0.957. The CS:DX:NH_4_PF_6_ system was found to decompose as the voltage goes beyond 1.5 V. Linear sweep voltammetry (LSV) revealed that the potential window for the most plasticized system is 1.5 V. The fabricated electrochemical double-layer capacitor (EDLC) was analyzed with cyclic voltammetry (CV) and charge-discharge analysis. The results from CV verify that the EDLC in this work holds the characteristics of a capacitor. The imperative parameters of the fabricated EDLC such as specific capacitance and internal resistance were found to be 102.9 F/g and 30 Ω, respectively. The energy stored and power delivered by the EDLC were 11.6 Wh/kg and 2741.2 W/kg, respectively.

## 1. Introduction

The implantation of natural polymers instead of synthetic polymers is an effective way to reduce plastic waste pollution [1,2]. Low cost, safety, portability, excellent light weight, flexibility, and good thermal stability are some of the unique aspects of solid polymer electrolytes (SPEs) [3]. Dextran (DX) is a natural polymer produced as a result of leuconostoc mesenteroides bacteria fermentation [4]. DX polymer is widely applied in the medical industry as a drug carrier [5]. The DX polymer chain consists of 1,6-α-D-glucopyranosidic linkages, where various oxygen including functional groups in this structure are very useful for ionic conduction [6]. With these interesting characteristics, a polymer electrolyte can be made out of it [7]. The inclusion of 20 wt.% ammonium nitrate (NH_4_NO_3_) into a DX host increased the conductivity from (8.24 ± 0.31) × 10^−11^ to (3.00 ± 1.60) × 10^−5^ S/cm [8]. A DC ionic conductivity of (1.67 ± 0.36) × 10^−6^ S/cm was acquired with 20 wt.% ammonium bromide (NH_4_Br) in the DX matrix [9]. The next polymer used in this work is chitosan (CS), which is extracted mainly from crustaceans—e.g., prawn, lobster, crawfish, and crabs [10]. CS has a β-(1→4)2-amino-2-deoxy-D-glucose-(D-glucosamine) structure, which enables ions to be transported from one electrode to another [11]. Rani et al. [12] reported that the addition of 40 wt.% NH_4_NO_3_ into a CS:cellulose-blend host provided a conductivity of 1.03 × 10^−5^ S/cm.

An electrolyte film and two porous electrodes are encompassed in the electrochemical double-layer capacitor (EDLC) device. Typically, carbon-based electrodes are used for generating supercapacitors (SCs) [13]. The different carbon-based electrode materials used in EDLCs are carbon aerogels, activated carbon (AC), carbon nanotubes, nanosized carbon, graphites, and carbon nanofibers [14]. The surface area of the electrode can expressly influence the performance of an EDLC device [15]. The huge surface area can offer adequate space for the ions to diffuse easily in the pores of an electrode and establish an ion–electron double layer [15]. Activated carbon (AC) is the most used activate material in EDLC applications because of its large surface area, easy preparation, and cost-effectiveness. The surface area of AC is up to ~ 2500 m^2^/g. As the mechanism of the energy storage of EDLC devices is through anon-Faradaic or adsorption/desorption, a high surface area electrode is highly demanded. DX:NH_4_Br-based EDLC has been reported to be stable for up to 100 cycles at ~ 2.05 F/g [9]. Based on our previous report [16], EDLC with the DX:NH_4_NO_3_:glycerol system possesses a specific capacitance of 15.7 F/g. Based on the work by Shukur et al. [17], EDLC with CS and NH_4_Br has a specific capacitance of 7.5 F/g. Shuhaimi et al. [18] managed to get a specific capacitance ranging from 13 to 18.5 F/g with ammonium salt:CS complexes. All the mentioned works regarding DX and CS have shown a quite low value of specific capacitance, which could be due to a low number of free ions. Electrolytes play an important role in EDLC applications; thus, various approaches such as plasticization and polymer blending can be used to enhance the performance of an EDLC.

The addition of plasticizer can enhance the number of free ions. The capacitance of an EDLC is dependent on the adsorption/desorption of free ions at the surface of the electrodes. Glycerol is one of the most used plasticizers when it comes to biopolymers because of their compatibility. The chemical structure of glycerol is C_3_H_8_O_3_, where oxygen atoms act as extra pathways for an ion to move from the electrolyte to the electrodes [19]. The presence of glycerol in the electrolyte improves the ion dissociation due to its high dielectric constant of 42.5 [20]. Prior works [21,22,23] reported a preliminary study on polymer electrolytes incorporating a limited amount of NH_4_PF_6_ up to 10 wt.% and studied the effect of plasticizer and filler addition. In their studies, they concluded that a high concentration of NH_4_PF_6_ is not preferable. In our previous work [24], we observed that ammonium salts with a low lattice energy below 600 KJ/mol are not desired for polymer electrolyte preparation due to the high rate of ion association. In this study, the enhancement of the free ion concentration was attempted with the help of glycerol plasticizer. Other than plasticization, polymer blending is another easy method that can be used to improve the ionic dissociation as well as transportation [25]. Polymer blending is when two or more polymers are mixed. The polymer blending technique produces an opportunity to create new materials that have characteristics that could not be obtained by a single polymer. The ion transport mechanism is easier in blended polymer, since more functional groups are available in the presence of two or more polymers [26]. Based on our previous work [27,28], the amorphous structure was noticeably enhanced in 40 wt.% DX and 60 wt.% CS. In this work, CS is blended with DX to provide more complexation sites. Ammonium hexafluorophosphate (NH_4_PF_6_) and glycerol are used as the ion provider and plasticizer, respectively. The aim of the present work is the preparation and characterization of proton (H^+^)-conducting polymer electrolyte membranes and their applications in proton EDLC devices. The plasticizer was introduced to the CS:DX:NH_4_PF_6_ to enhance both the ion transportation and salt dissociation. Finally, the highest conducting sample will be employed in the fabrication of the EDLC.

## 2. Experimental

### 2.1. Materials and Samples Preparation

High molecular weight CS (average molecular weight in the range from 310,000 to 375,000) and DX powders (average molecular weight in the range from 35,000 to 45,000) were utilized as the raw materials acquired by Sigma-Aldrich (St. Louis, MO, USA). For the polymer blend fabrication based on CS: DX, 40 wt.% (0.4 g) DX and 60 wt.% (0.6 g) CS were dissolved separately in 50 mL of 1% acetic acid at an ambient temperature for 90 min. These solutions were then combined and stirred for 3 h to acquire a homogeneous solution. A fixed amount of 40 wt.% (0.666 g) of NH_4_PF_6_ was included to the CS:DX solutions, and then different concentrations of glycerol with 14 (0.271 g), 28 (0.647 g), and 42 wt.% (1.206 g) were added separately with constant stirring to provide plasticized CS:Dex:NH_4_PF_6_: glycerol electrolytes. The plasticized electrolytes were labeled as CSDNHP1, CSDNHP2, and CSDNHP3 for CS:Dex:NH_4_PF_6_ and inserted in 14, 28, and 42 wt.% of glycerol, respectively. Afterward, the solutions were cast in Petri dishes and then were dried at an ambient temperature to create films. The films were inserted in a desiccator for better drying to produce films without solvent.

### 2.2. Structural, Morphological, and Impedance Characterizations

For structural examination, XRD spectra were acquired using a D5000 X-ray diffractometer (λ = 1.5406 Å) (Malvern Panalytical Ltd., Malvern, UK). The 2θ angle ranged between 10° and 80° with a resolution of 0.1°. The surface morphology and structural properties of the prepared blend electrolyte films were investigated by field emission scanning electron microscopy (FESEM) (FEI Quanta 200 FESEM, FEI Company, Hillsboro, OR, USA).

HIOKI 3532–50 LCR HiTESTER (Hioki, Nagano, Japan) was performed to study the electrical impedance spectroscopy (EIS) of the films in the frequencies from 50 Hz to 5 MHz. The prepared samples were cut into 2 cm diameter circles and placed between two stainless steel (SS) electrodes under spring pressure. The cell was connected to a computer program to provide real (Z′) and imaginary (Z″) parts of the spectra of the complex impedance (Z*).

### 2.3. Transference Number Analysis (TNM) and Linear Sweep Voltammetry (LSV)

The DC polarization method was used to obtain both the ion (*t*_ion_) and electron (*t*_elec_) transference numbers (TNMs). A V&A Instrument DP3003 digital DC power supply (V & A Instrument, Shanghai, China) was used in this analysis. The maximum conducting electrolyte was fixed between stainless steel (SS) electrodes to block the ions. The cell was applied with a voltage of 0.2 V at ambient temperature. TNM was employed to monitor the contributions of the ions and electrons to the overall conductivity and confirm the ionic conduction. LSV enabled us to see at what voltage the electrolyte decomposed. This analysis is very crucial for any energy device applications. The maximum conducting plasticized system was fixed between two SS electrodes and applied with a scan rate of 20 mV/s at an ambient temperature.

### 2.4. Fabrication and Characterization of EDLC

There were five steps in the electrode preparation. The first step was to prepare the carbon electrodes, where 0.25 g of carbon black was dry-combined with 3.25 g of activated carbon powder by a planetary ball miller (Changsha Yonglekang Equipment Co., ltd, Changsha, Hunan, China) at a 500 r/min rotational speed for 15 min. Secondly, 15 mL of N-methyl pyrrolidone (NMP) solvent was used to dissolve 0.50 g of polyvinylidene fluoride (PVdF) at room temperature. The third step was to dissolve the activated carbon-carbon black powders in the PVdF-NMP solution for 90 min. In the fourth step, the homogeneous solution was coated on aluminum foil and then dried in an oven for 120 min at 60 °C. The last step was to store the dried electrodes (thickness = 0.01 cm) in a desiccator filled with silica gel to remove extra moisture. The maximum conducting plasticized system was fixed between two activated carbon electrodes, which were cut into circles with areas of 2.01 cm^2^ and then inserted into CR2032 coin cells. The EDLC galvanostatic charge-discharge at a current density of 0.5 mA/cm^2^ was carried out by a NEWARE battery cycler. The Digi-IVY DY2300 Potentiostat (V & A Instrument, Shanghai, China) was used to examine the CV of fabricated EDLC at numerous scan rates ranging between 10 and 100 mV/s. The applied potential ranged between 0 and 0.9 V.

## 3. Results and Discussion

### 3.1. Structural and Morphological Analysis

**Figure 1:**
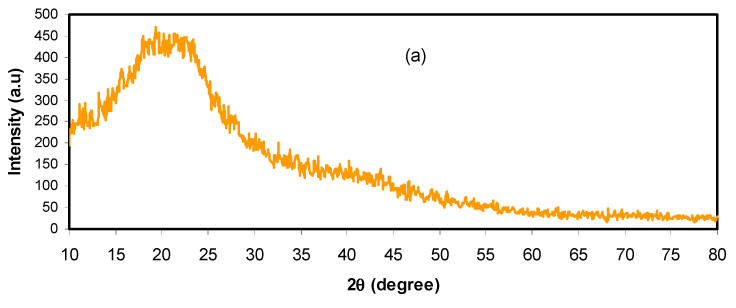

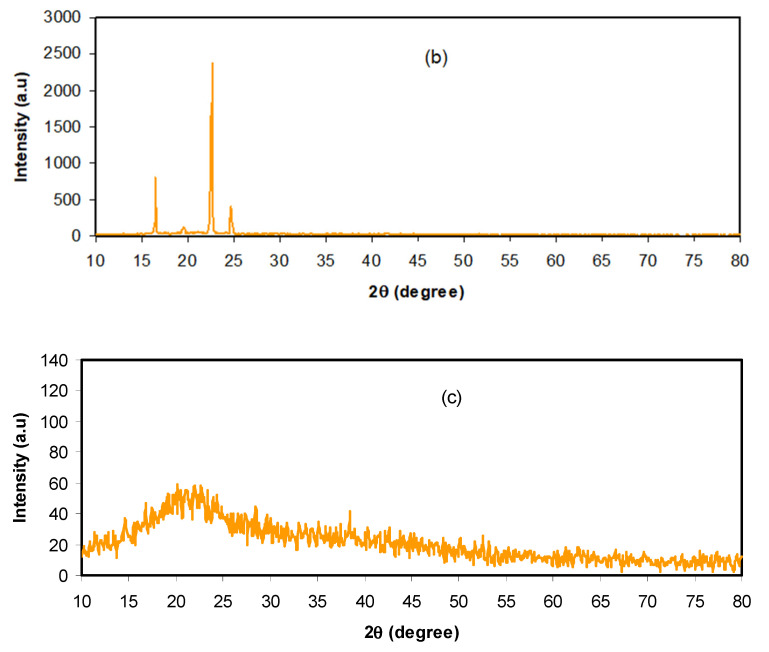
XRD spectra for the (**a**) pure chitosan (CS): dextran (DX), (**b**) CSDNHP1, and (**c**) CSDNHP3 electrolyte films.

XRD examination was performed for pure CS:DX and plasticized electrolytes at room temperature, as depicted in Figure 1. In earlier works, it was found that pure CS has shown various crystalline peaks at 2θ = 15.1°, 17.7°, and 20.9° [29,30], while DX has shown two hallows at 2θ = 18° and 23° [29,31]. Herein, the XRD of CS: DX has two hallows at low and high 2θ degrees, and no crystalline peaks are detected, as shown in Figure 1a. These wide hallows indicate a fully amorphous structure [32,33]. Previous studies reported that the broad peaks correspond to the amorphous structure of the electrolyte system [34,35,36]. In this research, it was determined that the hallow intensity of CS:DX:NH_4_PF_6_ roughly vanished with the insertion of 14 wt.% of glycerol, as illustrated in Figure 1b. The NH_4_PF_6_ salt is a very low lattice energy salt. The cations and anions of salts with very low lattice energies can be easily associated, and their protrusion to the sample surface produces these sharp peaks [37]. In our previous works, such crystalline peaks were not observed for NH_4_SCN, NH_4_I, and NH_4_F salts within the polymer blends [38,39,40]. The broad nature, which is evidence of the amorphous phase, was found to appear again at 42 wt.% of glycerol, as shown in Figure 1c. It is noteworthy that, at 42 wt.% of the incorporated plasticizer, these crystalline peaks vanished and the hallow intensity decreased compared to the pure CS: DX spectrum. According to the literature, the ionic transport in polymer electrolytes occurs principally in the amorphous phases, which is directed by the segmental motion of the polymer chains [41]. The results of the present work establish that plasticization is one of the most adopted approaches used to suppress the crystallinity of polymer electrolytes. The glycerol plasticizer can dissociate more salts and disrupt the hydrogen bonding between polymer chains [41]. Thus, this improves the overall amorphous phase of the prepared samples, which acts as a pathway for ion conduction [41]. Additionally, more free ion will be available for conduction. These data results validate the electrolytes’ amorphous structure. The amorphous structure offers larger ionic diffusivity, leading to a higher DC conductivity. Impedance study in later sections may give more information. No peaks of NH_4_PF_6_ salt were emerged in the CS: DX electrolyte system, which shows the entire salt dissociation in the electrolyte system.

**Figure 2:**
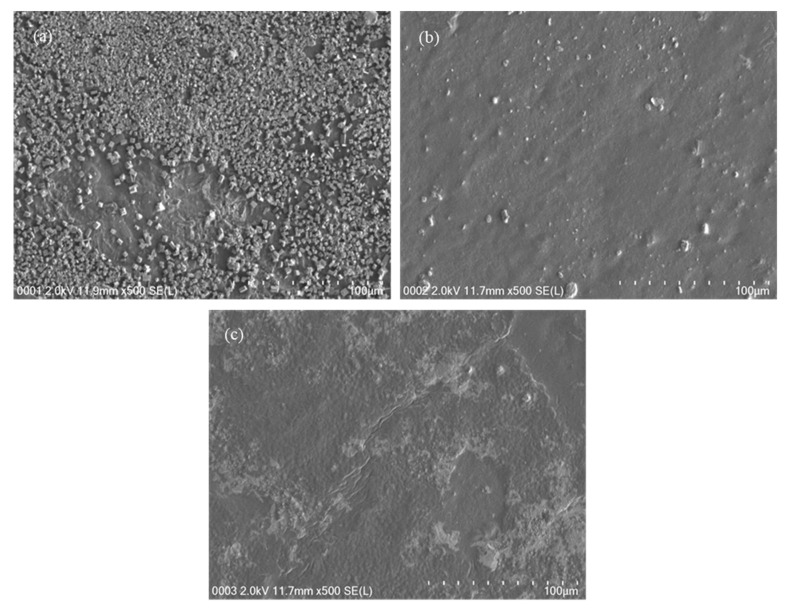
FESEM images for the (**a**) CSDNHP1, (**b**) CSDNHP2, and (**c**) CSDNHP3 electrolyte films.

Field emission scanning electron morphology (FESEM) is a useful technique for investigating the surface morphology of various polymer electrolytes. Figure 2a–c present the FESEM micrograph images of plasticized CS:DX blend films. The FESEM image of the CSDNHP1 system shows many white aggregates due to ion association at a low concentration of glycerol plasticizer. These white particles have reduced the ion concentrations that play a vital role in ion conducting-based electrolyte. In our previous studies, we observed many white particles on the surface of CS:PEO and CS:PS incorporating NH_4_BF_4_ slat due to a high association of ions [24,37]. Clearly, with increasing the glycerol concentration to 28 wt.% (see Figure 2b), the number of ion aggregates is reduced and thus more salts can be dissolved in the proximity of the plasticizer. The FESEM image of CSDNHP3 looks smooth and uniform, which is a good sign of reducing ion aggregates and thus increasing the number of free ions for conduction.

### 3.2. Impedance Study

**Figure 3:**
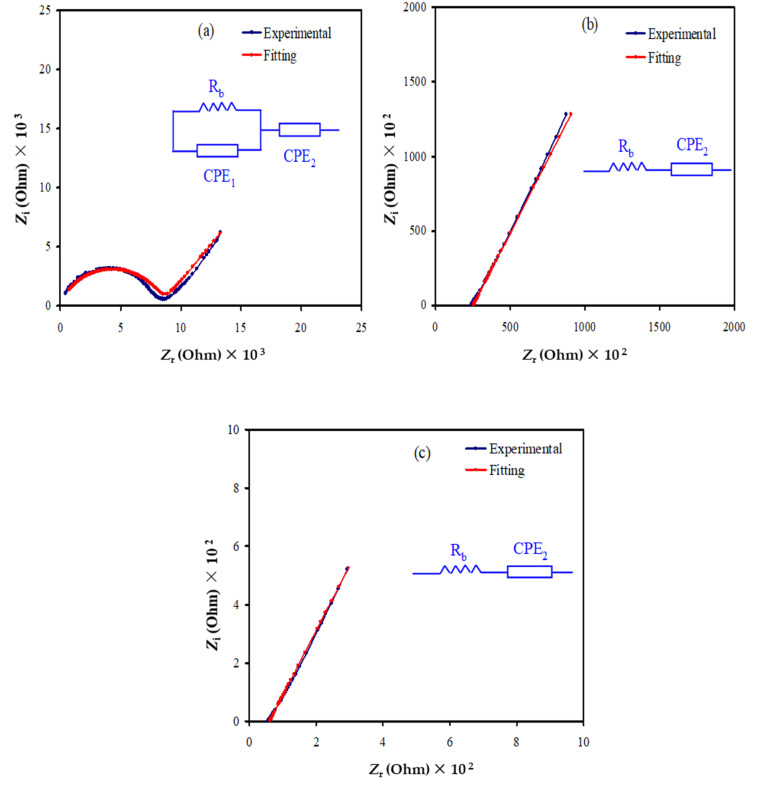
Electrochemical impedance spectroscopy (EIS) plots for the (**a**) CSDNHP1, (**b**) CSDNHP2, and (**c**) CSDNHP3 electrolyte films.

Electrochemical impedance spectroscopy (EIS) is a new and influential method in the study of the electrical properties of polymer electrolytes and the interfacial region between electronically conducting electrodes [42,43]. The impedance plots for CS: DX: NH_4_PF_6_: glycerol systems at ambient temperature are indicated in Figure 2a–c. The electric impedance plots indicate two main regions: a semicircle appeared at the high frequencies, owing to the electrolyte bulk character, and a linear tail emerged at the low frequencies, which are associated with the blocking electrodes [44,45]. It is noticeable in Figure 3a–c that, with rising glycerol content, the bulk resistance (*R_b_*) is lowered. The values of *R_b_* are measured at the point where the semicircle intercepts the real axis (Z_r_). Equation (1) is employed to measure the films’ DC conductivity based on the *R_b_* values and the films’ dimensions:

(1) $$\sigma_{dc}=\;\left[\frac{1}{R_{b}}\right]\;\times \;\left[\frac{t}{A}\right]$$

In Equation (1), the thickness of the films and the surface area of the electrodes are correspondingly referred to by *t* and *A*. Table 1 lists the DC conductivity for each film. The highest conductivity of the plasticized electrolyte system is promising for application in an EDLC device. Former studies discovered that polymer electrolytes with conductivity series from 10^−5^ to 10^−3^ S/cm are useful for electrochemical device applications [9,16,17,28,32]. Therefore, the conductivity (3.06 × 10^−4^ S/cm) realized in the existing study is a guarantee for ion-conducting device application. The outcomes designate that plasticized ion-conducting electrolytes display a high conductivity in association with raw CS conductivity (1.73 × 10^−10^ S/cm) [33]. Our prior study also discovered that neat CS:DX had a low DC conductivity (5.01 × 10^−10^ S/cm) [28]. More insights regarding the electrolytes’ electrical properties are obtained from the impedance plot modeling by electrical equivalent circuits (EECs). It is possible to measure the circuit elements and bulk resistance through the impedance plots’ modeling.

The EEC model is normally engaged in impedance spectroscopy inspection, as the EEC model is swift, simple, and creates an entire picture of the systems [46,47]. The EIS plots are expressed in terms of the EEC containing *R_b_* for the carrier species in the films, as well as two constant-phase elements (CPEs), which are CPE1 and CPE2, as interpreted in the inset of Figure 3a–c. The CPEs are non-ideal capacitors. The region of high frequencies displays the connection of *R_b_* and CPE1 (capacitor 1) in parallel, whereas the region of low frequencies (slanted line, a non-ideal capacitor, CPE2 in series) displays the created double layer capacitance among the SPE and electrodes. The CPE is generally employed in EEC rather than an ideal capacitor in real systems. The impedance of CPE *(Z_CPE_*) is expressed as [48]:

(2) $$Z_{CPE}=\frac{1}{C\omega^{p}}\left[\cos\left(\frac{\pi p}{2}\right)-i\sin\left(\frac{\pi p}{2}\right)\right].$$

In Equation (2), *C* refers to the CPE capacitance, *ω* refers to the angular frequency, and *P* relates to the vertical axis deviation of the impedance plots. The real part *(Z_r_*) and imaginary part (*Z_i_*) of the complex impedance (Z^*^) associated with the EEC (inset of Figure 2a) are written as:

(3) $$Z_{r}=\frac{R_{b}^{2}(A1)+R_{b}}{2R_{b}(A1)+R_{b}^{2}C_{1}^{2}\omega^{2p1}+1}+\frac{A2}{C_{2}\omega^{p2}},$$

where $A1=C_{1}\omega^{p1}\cos\left(\frac{\pi p_{1}}{2}\right)$ and $A2=\cos\left(\frac{\pi p_{2}}{2}\right)$,

(4) $$Z_{i}=\frac{R_{b}^{2}(A3)}{2R_{b}(A1)+R_{b}^{2}C_{1}^{2}\omega^{2P1}+1}+\frac{A4}{C_{2}\omega^{p2}},$$

where $A3=C_{1}\omega^{p1}\sin\left(\frac{\pi p_{1}}{2}\right)$ and $A4=\sin\left(\frac{\pi p_{2}}{2}\right)$.

In Equation (3), *p*_1_ relates to the deviation of the semicircle radius from the imaginary axis and *p*_2_ relates to the deviation of the tail/spike from the real axis. C_1_ refers to the capacitance at high frequency and C_2_ refers the capacitance at low frequency.

All the parameters of the circuit elements, which are used for impedance plot fitting for each sample, are listed in Table 2. In the Cole–Cole plots, at higher glycerol amounts (Figure 3b–c) the semicircle is missed, meaning that just the resistive component of the electrolyte systems exists [49]. In such cases, the *Z_r_* and *Z_i_* related to the EEC are written as:

(5) $$Z_{r}=R+\frac{A2}{C_{2}\omega^{p2}},$$

(6) $$Z_{i}=\frac{A4}{C_{2}\omega^{p2}}.$$

In Table 2, *K*_1_ and *K*_2_ are the reciprocals of capacitance at high and low frequencies, respectively.

### 3.3. Transference Number Analysis (TNM)

**Figure 4:**
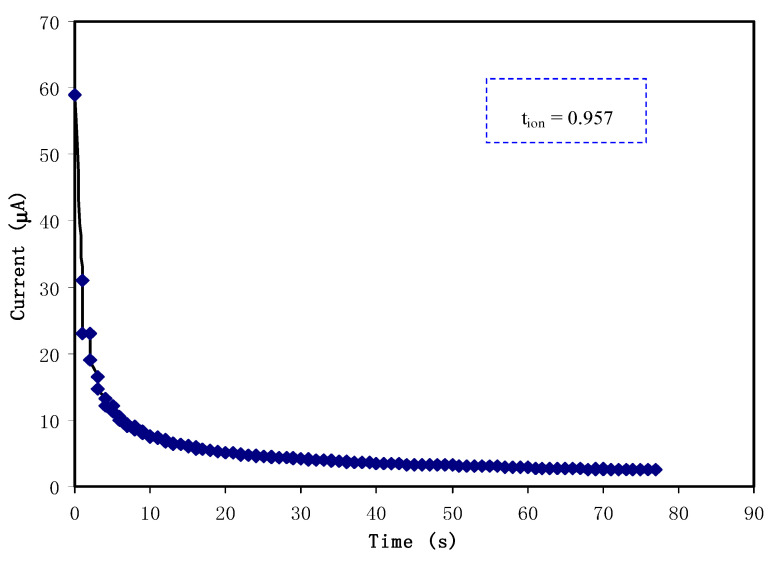
Polarization of the highest conducting electrolyte at 0.2 V.

Typically, in polymer electrolytes the conductivity is due to the contribution of both electrons and ions. For EDLC applications, *t*_ion_ must be larger than *t*_elec_*;* thus, TNM analysis has been performed at an operating voltage of 0.2 V. Ions are blocked at the stainless steel (SS) electrode surface. The polarization of SS | highest conducting sample | SS is shown in Figure 4. A large value of current can be seen at the beginning of the polarization, which is 58.9 μA. At this point, both ions and electrons are conducted. At 5 s, the current drops to 10 μA. At this stage, some of the ions from the electrolyte are blocked at the surface of the SS electrodes and form a charge double layer. Beyond 40 s, the stabilization of current value can be seen, where it becomes constant at 2.4 μA. The cell is completely polarized at this point, and only electrons can pass through the SS electrode. This pattern of the current value is normal for ionic conductors [50]. *t*_ion_ can be obtained via the following equation:

(7) $$t_{ion}=\frac{I_{i}-I_{ss}}{I_{i}},$$

where *I_i_* and *I_ss_* are the current at the beginning and in steady state, respectively. *t*_elec_ can be obtained as the summation of *t*_ion_, and *t*_elec_ is equal to 1. The contribution of ions to the overall conductivity in this work is found as 0.957, while the electron is 0.043. We discovered that *t*_ion_ is bigger than *t*_elec_, thus ions are confirmed to be the dominant charge carriers in the overall conductivity. Rani et al. [51] stated that good polymer electrolytes must have a *t*_ion_ > 0.90. Thus, the highest conducting plasticized system in this work has reached one of the requirements to be applied in EDLC applications.

### 3.4. Linear Sweep Voltammetry (LSV)

**Figure 5:**
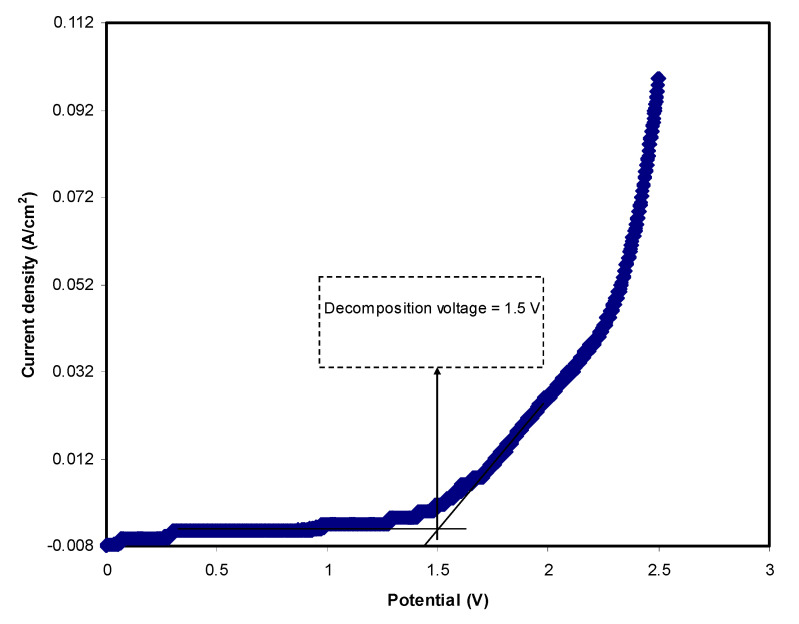
LSV plot of the highest conducting plasticized system at 20 mV/s.

In energy devices studies, electrolyte electrochemical stability is an important parameter to obtain. The maximum potential limit of the electrolyte can be analyzed using LSV analysis. Figure 5 portrays the LSV for SS | highest conducting sample | SS at 20 mV/s. The current is noted to be stable at potentials of less than 1.5 V. As the potential increases to 1.5 V, the current starts to rise considerably. This is recognized as the decomposition voltage of the electrolyte. This value is adequate for the application of a proton-based energy storage device; typically, an electrolyte should have at least a decomposition voltage of 1 V.

### 3.5. Cyclic Voltammetry (CV)

**Figure 6:**
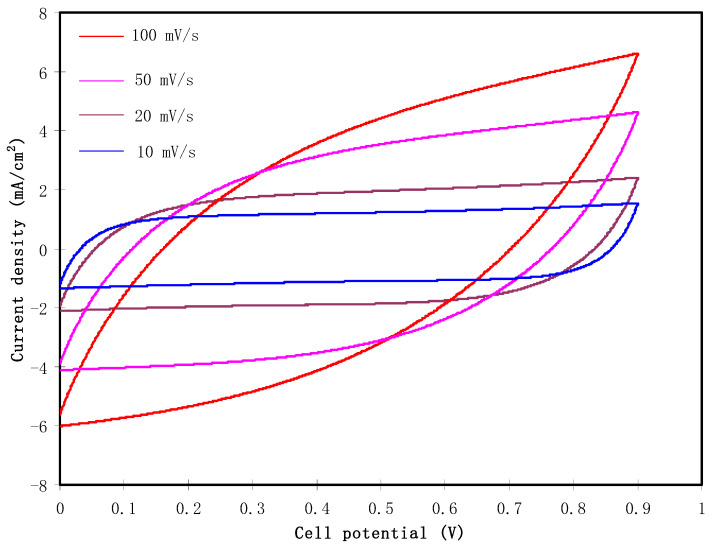
Cyclic Voltammetry (CV) plot of the fabricated EDLC at various scan rates.

The fabricated EDLC must undergo CV analysis before the charge-discharge process. CV analysis is used to check the existence of capacitive behavior in the electrolyte, as well as finding the specific capacitance of the EDLC. Figure 6 shows the CV plot of the fabricated EDLC at scan rates of 100, 50, 20, and 10 mV/s. It is observed that for each scan rate, the CV profiles show no redox peak. The redox peak indicates deintercalation/intercalation or Faradaic processes that are entirely dissimilar to the mechanism of energy storage in the EDLC. The EDLC will accumulate energy within adsorption and desorption or non-Faradaic processes [52,53,54]. The pattern of the CV has a leaf-like shape at 100 mV/s. The shape turns to a more rectangular shape as the scan rate decreases to 10 mV/s. One of the features of a capacitor is that the response of CV is dependent on the scan rates. The conduction of ion happens at a fast rate at a high scan rate. Furthermore, due to the internal resistance and carbon porosity, the current dependence on voltage is produced [55]. The specific capacitance (*C_cv_*) is expressed as:

(8) $$C_{cv}=\int_{V_{l}}^{V_{f}}\frac{I\left(V\right)dV}{2mx\left(V_{f}-V_{i}\right)}.$$

Here, *I(V)dV* refers to the CV area plot that is calculated using the Origin 9.0 software by the integration function. *x* and *m* are the scan rate and the activated carbon mass, correspondingly. *V_i_* is the lower voltage, which is 0 V, and *V_f_* is the upper voltage, which is 0.9 V. The values of *C_cv_* obtained are 23.1, 41.2, 65.8, and 87.8 F/g for a scan rate of 100, 50, 20, and 10 mV/s, respectively. Ions are conducted at a constant rate when a low scan rate is used, thus it has developed a greater charge double layer. At high scan rates, ions are moved at a quick rate. This condition reduces the development of the charge double-layer, which drops the capacitance value [56]. The reduction in *C_cv_* with an increase in the scan rate is related to the internal resistance presence. The current time scale to achieve a horizontal stable value on the scan reversal of potential will increase at larger scan rates. The more delay at the switching potential will induce the deceleration reformation of the double layer as a result of the great resistance of ion mobility in the pores of activated carbons of the EDLC [57,58].

**Figure 7:**
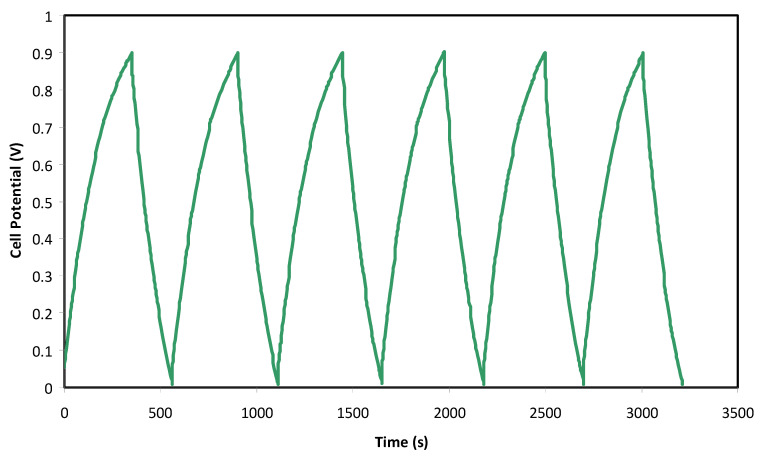
Charge-discharge profiles of the fabricated EDLC at selected cycles.

Figure 7 illustrates the common charge-discharge profile of the EDLC at selected cycles. The EDLC is applied to a current density of 0.5 mA/cm^2^ from 0 to 0.9 V for 200 cycles. Perfect linearity behavior cannot be observed at the slope of the charge and discharge part. This may be related to the occurrence of some electrochemical reactions at the electrode/electrolyte interface and thus give rise to the potential drop and effect on the charge-discharge profile. A very small reaction at the interface of the electrode and electrolyte may result in a small pseudo capacitance, which might also affect the linearity behavior of the charge-discharge pattern. A nearly linear discharge slope indicates that the EDLC has a suitable capacitor energy storage process [59]. A normal EDLC usually has a slight drop in potential value before the charge and discharge processes. This is because of numerous causes, including the gap between the electrolytes and current collectors, as well as the electrolyte bulk resistance. Specific capacitance (*C_spe_*) can be obtained from the discharge part in Figure 6 via Equation (9):

(9) $$C_{spe}=\frac{i}{ms}$$

Here, *i* refers to the applied current, *s* refers to the discharge part of the slope, and *m* is the mass of active material.

**Figure 8:**
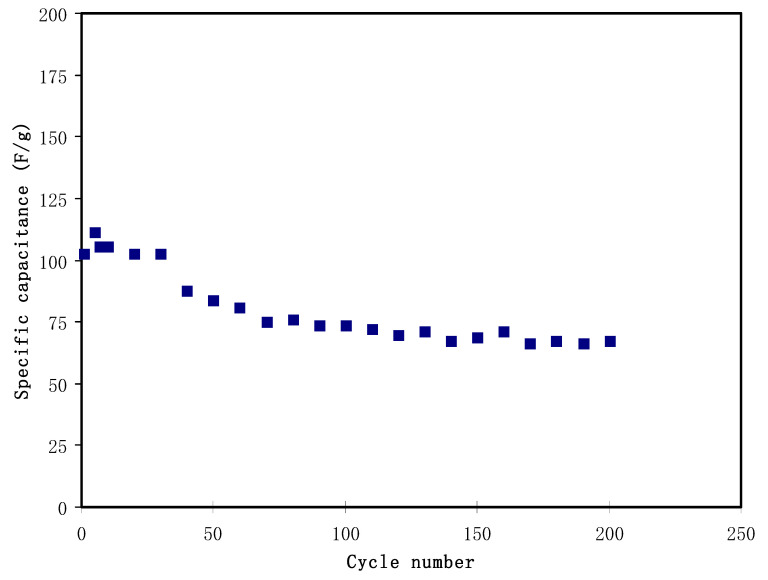
Specific capacitance of the EDLC at 0.5 mA/cm^2^ for 200 complete cycles.

Figure 8 shows the plot of the *C_spe_* against the cycle number. The *C_spe_* at the 1st cycle is 102.9 F/g, which then reduces to 80.7 F/g at the 60th cycle. The reduction could be due to several charges, which are consumed in an irreversible mechanism with a weakly bound functional group—for example, OH^−^ groups absorbed on the surface of the electrodes. Other than that, some pores of the electrodes could be permanently blocked after several charge-discharge processes [60]. The *C_spe_* stabilization is observed from the 60th to the 200th cycles, with an average of 70.4 F/g. This value is in the range of *C_spe_* obtained from the CV analysis. The existence of glycerol creates more differing pathways for the transportation of ions because of the abundance of OH^−^ groups. Plasticizer improved the free ion density. These two circumstances encourage additional free ions at the surface of the electrodes, thus enhancing the specific capacitance. The continuous decrease in the *C_spe_* with the increasing cycle number may be related to the lattice energy of the NH_4_PF_6_ salt. The *U_L_* for NH_4_PF_6_ was calculated using Kapustinskii’s equation [61]:

(10) $$U_{L}=\frac{1202(v)(Z^{+})(Z^{-})}{d_{o}}(1-\frac{0.345}{d_{o}}).$$

Here, *v* refers to the number of the ions, *d_o_* refers the sum of the anion and cation radii, and *Z^+^* and *Z*^−^ refer to the charge number. The NH_4_PF_6_ lattice energy is 543.06 kJ/mol. Salts with a low lattice energy can be more simply dissociated compared to the higher lattice energy ones [24]. This easy salt dissociation will result in providing a larger number of mobile ions and give rise to the overall capacitance for the given polymer electrolyte. However, these free ions in salts with a low lattice energy such as NH_4_PF_6_ have a great tendency for recombination [24], as noted in the current work, where many crystalline peaks are observed (see Figure 1b) due to the salt recrystallization. This is basically correlated to the fact that the electrostatic force among functional groups in polymers and cations is smaller than that present amongst anions and cations, and, as a result, further agglomerated ions will emerge on the film surface. In this recombination process, the radius anions plays a crucial role [24]. In this study, the PF_6_^−^ anion has radius of 254 pm [62], which is bigger than other anions such as SCN^−^ (213 pm) [63], I^−^ (206 pm), or NO_3_^−^ (185 pm) [64]. These larger ions (PF_6_^−^) have a lesser mobility and can be easily associated with the cations. This might be the reason for the lesser steady values of EDLC capacitance with NH_4_PF_6_ salt in this work. It is clear from the capacitance plot (Figure 7) that, with the increasing cycle number, the EDLC performance decreased and at the same time the ESR value (see Figure 8) increased. The possible explanation for this behavior of the EDLC cell is the low value of the lattice energy of NH_4_PF_6_. This means that with the elapsing of time more ions may associate, which reduces the number of free ions available for conduction and, thus, the charge storage decreases [65].

The equivalent series resistance (*ESR*) is another important characteristic which needs to be known to investigate the EDLC internal resistance. The *ESR* of the EDLC is obtained using Equation (11):

(11) $$ESR=\frac{V_{r}}{i}.$$

**Figure 9:**
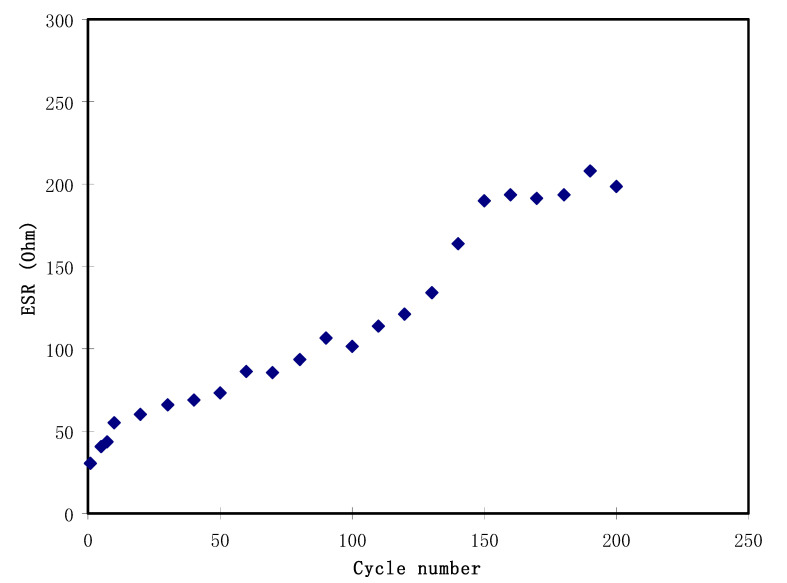
The equivalent series resistance (ESR) of the EDLC at 0.5 mA/cm^2^ for 200 complete cycles.

It is noticeable in Figure 7 that there is a slight reduction in voltage (*V_r_*) before each of the discharging processes. The reduction in voltage is in the range between 0.03 and 0.2 V, which is due to the internal resistance built up in the EDLC. Figure 9 shows the *ESR* of the EDLC for 200 cycles. The *ESR* of the EDLC ranged from 30.0 to 207.0 Ω. A small value of *ESR* indicates an excellent electrode-electrolyte contact, which means it is easier for ions to move from the bulk electrolyte to the surface of the electrodes and undergo polarization [66]. The internal resistance results from the active material resistances, connectors, and connector and polymer electrolyte bulk resistance [67]. Kumar and Bhat [68] monitored such increment in voltage drop during charge-discharge cycles that caused an increment in *ESR* as a result of the electrolyte in the EDLC.

The amount of energy that an EDLC can hold is called specific energy (*E*), and it can be expressed as:

(12) $$E=\frac{1}{2}(C_{spe}V^{2}).$$

**Figure 10:**
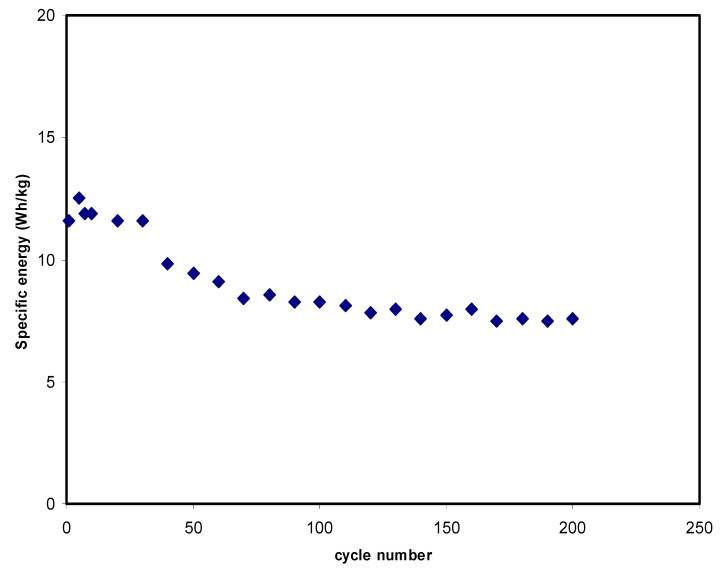
Specific energy of the EDLC at 0.5 mA/cm^2^ for 200 complete cycles.

The value of *E* calculated is depicted in Figure 10. The *E* at the 1st cycle is 11.56 Wh/kg in Figure 9. The *E* values then drop for only some cycles, and after that they are almost stable at 7.9 Wh/kg on average from the 70th to the 200th cycle. At this stage, ions are assumed to experience the same energy barrier as it moves from the electrolyte to the electrode surfaces [69]. This result infers that the EDLC electrochemical stability happens to be more constant upon the 70th cycle of charge-discharge mechanisms. The decrease in these electrochemical characteristics—e.g., capacitance and energy density—is attributed to the electrolyte depletion. The decrement in *E* during the cycle number is mostly caused by the increment in the internal resistance, which leads to the energy loss increment within the mechanism of charge and discharge cycles. During the swift charge-discharge mechanism, some ions are re-associated back to provide ion pairs or triplets, which blocks the free ion transportation [70].

The amount of energy that an EDLC can delivers is called specific power. Specific power (*P*) can be calculated if the value of the *ESR* of each cycle is known and can be obtained via the following equation:

(13) $$P=\frac{V^{2}}{4m(ESR)}$$

**Figure 11:**
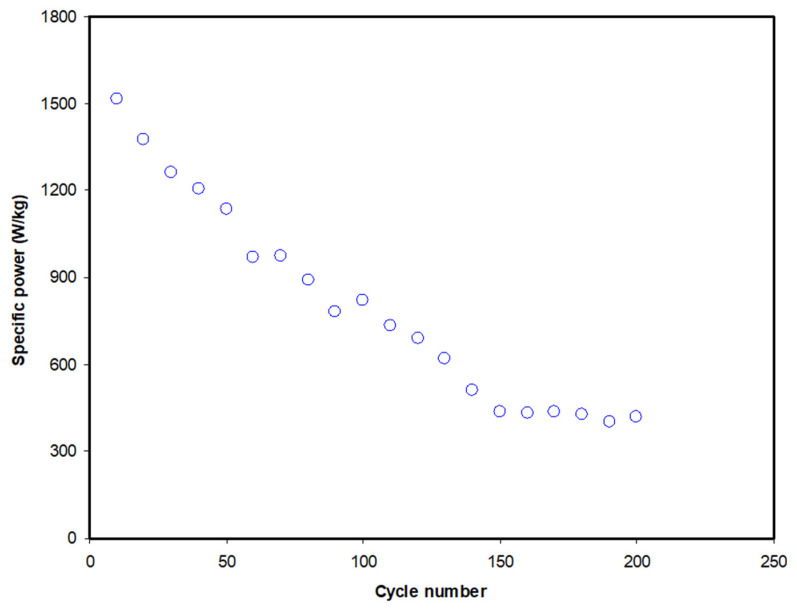
Specific power of the EDLC at 0.5 mA/cm^2^ for 200 complete cycles.

The first 10 cycles of the EDLC demonstrate a decrease of 44.7% from 2741.2 to 1515.2 W/kg. The value of *P* experiences another reduction to 510.0 W/kg as the cycle number increases to 140. *P* is then observed to be stabilized beyond the 150th cycle with an average of 425.8 W/kg. By referring to the equation of *P*, we can see that it is strongly related to the value of *ESR*. It is noticeable that the pattern of *ESR* in Figure 9 is the reverse of the pattern of *P* in Figure 11. This indicates that an increase in internal resistance affects the energy delivery process, thus reducing the specific power.

## 4. Conclusions

A protonic-based polymer electrolyte was synthesized with a solution cast technique where ammonium hexafluorophosphate (NH_4_PF_6_) and glycerol act as the ionic source and plasticizer, respectively. The XRD method displayed that the plasticized electrolyte system with the highest value of DC conductivity was the most amorphous system. It was found that the ions in CS:Dex:NH_4_PF_6_:glycerol were the main carriers, as the outcome from the TNM examination showed that *t*_ion_ > *t*_elec_. The potential widow of the most plasticized polymer electrolyte in this research was steady until 1.5 V, which verified that it is utilized in the EDLC construction. The capacitive characteristic of the EDLC was confirmed with a CV study, as no oxidation and reduction peaks were examined. The specific capacitance was identified to be impacted by the scan rates applied. The EDLC’s important parameters were monitored to 200 cycles. The specific capacitance, ESR, energy, and power densities were 102.9 F/g, 30 Ω, 11.6 Wh/kg, and 2741.2 W/kg, respectively. These obtained results are of special importance, since they open the gate to the commercialization of biodegradable polymer electrolytes in the field of energy devices. However, more efforts are necessary to enhance the conductivity, electrochemical stability, compatibility, and electrode/electrolyte contact of the biodegradable polymer electrolyte to meet the industrial level. These parameters could be tuned and further enhanced to achieve a high cyclic and high rate performance of EDLC by carefully selecting the host blend polymer and the salt, then introducing appropriate plasticizer and nano-fillers, thus modifying the electrodes in future works.

## Figures and Tables

**Table 1:** **Direct current** (DC) conductivity for the plasticized electrolyte system at room temperature.

Designation	Rb (Ohm)	Conductivity (S cm^−1^)
CSDNHP1	0.88 × 10^4^	2.02 × 10^−6^
CSDNHP2	3.3 × 10^2^	5.38 × 10^−5^
CSDNHP3	0.59 × 10^2^	3.06 × 10^−4^

**Table 2:** The electrical equivalent circuits (EEC) fitting parameters for the CS:DX:NH_4_PF_6_: glycerol systems at room temperature.

Sample	*K*_1_ (F^−1^)	*K*_2_ (F^−1^)	*C*_1_ (F)	*C*_2_ (F)	*P1*	*P2*
CSDNHP1	4 × 10^8^	2.1 × 10^5^	2.5 × 10^−9^	4.76 × 10^−6^	0.79	0.57
CSDNHP2	-	8.08 × 10^4^	-	1.23 × 10^−5^	-	0.70
CSDNHP3	-	3.88 × 10^4^	-	2.57 × 10^−5^	-	0.73
